# Evolution, composition and functions of cullin E3 ubiquitin ligases in trypanosomes

**DOI:** 10.1038/s41598-025-32077-9

**Published:** 2025-12-18

**Authors:** Ricardo Canavate del Pino, Martin Zoltner, Erin R. Butterfield, Mark C. Field

**Affiliations:** 1 School of Life Sciences, University of Dundee, Dundee, UK; 2 Department of Parasitology, Charles University in Prague, Faculty of Science, Vestec, 252 42 Czech Republic; 3 Institute of Parasitology, Biology Centre, Czech Academy of Sciences, České Budějovice, Czech Republic; 4 Sygnature Discovery, The Discovery Building, BioCity, Pennyfoot Street, Nottingham, NG1 1GR UK

**Keywords:** Cullin, Ubiquitin, E3 ligase, Protein turnover, Evolution, Affinity isolation, Cryomilling, Eflornithine, Ornithine decarboxylase, Trypanosoma, Proteomics, Biochemistry, Computational biology and bioinformatics, Microbiology

## Abstract

Post-translational modifications (PTMs) modulate protein functions, with ubiquitylation a pre-eminent example, and playing major roles in protein turnover. Ubiquitylation utilises a ligase enzyme cascade for conjugation of ubiquitin to client proteins, of which there are a large number in humans and lesser numbers in unicellular eukaryotes. The Cullin-RING ligases are amongst the most complex ligase subfamily and are present across the eukaryote lineage. We have reconstructed the evolution of cullin-RING E3 ubiquitin ligases across eukaryotes and experimentally determined the composition of six of seven cullin complexes in trypanosomatids. We find considerable diversity within cullins and reconstruct at least four ancestral pan-eukaryotic subfamilies. Furthermore, we identify expansions of cullin client adaptor protein families, novel client adaptors and demonstrate client specificity in trypanosomatids. We also find evidence for increasing complexity within client adaptors, suggesting ongoing expansion of adapter architecture. Finally, we show that turnover of ornithine decarboxylase (TbODC), an important target of the trypanocide eflornithine, is mediated by TbCul-A/CUL-1. These studies highlight lineage-specific aspects of cullin E3 ligases and their contributions towards eukaryotic complexity.

**Supplementary Information:**

The online version contains supplementary material available at 10.1038/s41598-025-32077-9.

## Introduction

Maintaining a steady state or facilitating rapid changes to protein composition following altered conditions or developmental progression are essential for cellular viability. Of many mechanisms involved in controlling protein abundance, the attachment of ubiquitin, ubiquitylation, to client proteins is a prominent mechanism. Ubiquitylation has many roles, with decreased stability via proteasomal targeting and/or altered sub-cellular localisation as the major activity^1^. Initially, free ubiquitin is activated by an E1 ubiquitin transferase, followed by transfer via an E2-conjugating enzyme to an E3 ligase. The final step involves recognition, by the E3 ligase, of a client protein and the covalent attachment of ubiquitin via the C-terminal carboxyl to NH_2_ lysine side chains, creating an isopeptide bond^2^.

The vast majority of E3 ligases are classified into RING, HECT, U-box, PHD-finger or RING between RING (RBR) cohorts, based on domain architecture. *Homo sapiens* possesses over 600 RING, 30 HECT and 12 RBR proteins^3^, and amongst the RING class the ligases are notably diverse, characterised by a zinc finger RING domain and frequent recruitment into multisubunit complexes. Amongst these the *H. sapiens* SCF (Skp/cullin/F-box) is the prototypical cullin-RING complex and possesses a core cullin scaffold protein binding RBX1, a RING E3 ligase, at one terminus of the complex and SKP1/F-box client protein adaptors at the other^4,5^. A considerable repertoire of client adaptor subunits allows cullin complexes to recruit and recognise numerous clients, and ligase activity is tightly regulated via neddylation and phosphorylation^6,7^. The basis of this specificity, origins and adaptations of ubiquitylation pathways remain poorly characterised. Significantly, the anaphase promoting complex (APC) and replisome also contain a cullin/Rbx ubiquitin ligase at their core and govern turnover of critical proteins during mitosis^8,9^.

Originally considered eukaryotic-specific, ubiquitylation clearly predates eukaryogenesis^10,11^, and the identification and biochemical characterisation of a complete ubiquitylation pathway in Archaea bacteria is consistent with the near universal presence of the proteasome across evolution. Moreover, ubiquitylation systems have been identified in many bacterial lineages, many of which likely result from lateral gene transfer events^12^. However, the ubiquitylation system is considerably expanded and diversified in eukaryotes, with a repertoire of over 600, 500 and 100 E3 ligases in *H. sapiens, Arabidopsis thaliana* and *Saccharomyces cerevisiae* respectively. While ubiquitylation pathways are present in all major eukaryote lineages, systematic analysis has focused on animals, higher plants and fungi^3,13,14^. In silico comparative studies are currently limited, in part due to the complexity of the ubiquitylation machinery, but some patterns can be recognised. Specifically, expansions in RING E3 ligases are common, while HECT and RBR families are generally smaller; the latter are of similar sizes as the E1 and E2 enzyme families, with typically two to 40 paralogs respectively in most organisms^7,15^. However, the presence of lineage-specific components within ubiquitylation pathways remains largely unaddressed.

Trypanosomes are members of the Kinetoplastida within the Discoba lineage, and a model system for evolutionary cell biology, combining tractability, phylogenetic position close to the eukaryotic root, divergent biology and comparative simplicity in genome and proteome size. The Discoba represent an early eukaryotic branch and hence are valuable for illuminating early differentiation events following eukaryogenesis^16,17^. Unsurprisingly, ubiquitylation in trypanosomes regulates the cell cycle and is essential for cellular homeostasis^18–22^. Both the proteasome and ubiquitylation are involved in targeting and turnover of trypanosome surface proteins^23–26^, and a canonical ESCRT system functions in late endocytic trafficking^27–29^. Further, genetic screens implicate ubiquitylation in trypanosome stress responses and gene expression^30^. For example, key elements including E3 ligases are drug resistance-associated genes, with modes of action remaining to be determined^31^. Some trypanosomatids secrete and direct ubiquitylation-regulating proteins into the host cell nucleus, but again precise functions remain unknown^32^. Previous work addressed the potential functions of two trypanosomatid cullins, Cullin-1 and Cullin-5 (referred to here as TbCul-A and TbCul-E), although neither their composition nor their impact on the proteome were investigated^18,19^. Most recently a cullin complex from *Leishmania infantum* was described, a likely a homolog of Cullin-1 (or Cul-A); this complex is essential^33^.

Here we attempted to address the following questions: How have the cullin protein scaffolds evolved across eukaryotes, are there novel client adaptor subunits within a divergent organism such as trypanosomes, and can we infer functions via whole cell proteomics? To address these we used phylogenetic reconstruction to generate an in-depth view of cullin evolution and through single step immunopurification, characterised trypanosome cullin complex composition to identify lineage-specific adaptations and used RNAi to examine the functions of select cullins. We find considerable under-appreciated complexity within cullin ligases, suggesting inter-taxon variation and identify an unexpected role for a cullin complex in trypanosome drug sensitivity.

## Results

### Evolution of the cullin scaffold

Cullin-RING complexes are prominent amongst E3 ligases due to the large cohorts of adaptor subunits deployed for recruitment, recognition and transfer of ubiquitin to client proteins. Adaptor subunits recruit specific client proteins; both the RING E3 ligase and these adaptor subunits are physically connected through the cullin scaffold protein. The number of cullins in the genome varies between species, with seven in *H. sapiens,* six in *A. thaliana* and four in *S. cerevisiae,* each forming distinct complexes with specific families of substrate adaptors^34–39^. We extended previous analysis by embracing a taxonomically broader range of species, and including examples from the Excavata to capture early events in cullin evolution following eukaryogenesis.

Previous phylogenetic reconstruction of cullin evolution based on animals, fungi and plants identified three ancestral clades, Culα, Culβ and Culγ^35^. This ancestry is supported by specific client adaptor subunits associated with each complex; hence Culα clade members interact with several families of F-box proteins, Culβ with DCAF-domain-containing subunits and Culγ with BTB proteins. Significantly, kinetoplastids have been reported to encode between five and seven cullin genes, but evolutionary origins are unclear due to sequence divergence^18,33,40^, hampering accurate reconstruction.

We used ScrollSaw, a method for building a phylogeny from highly diverse sequences, for analysis of cullins^41^. This dataset included over 130 species and ~ 900 protein entries, including the anaphase promoting complex (APC) subunit 2, a highly conserved cullin-RING E3 ligase relative as outgroup. Phylogenies were generated for each eukaryotic supergroup and sequences with shortest branches selected for a pan-eukaryotic reconstruction. We named Excavata sequences as TbCul-A, -B etcetera to avoid implicit assumptions of orthology with other organisms (Table 1).

**Table 1:** Summary of protein interactions identified from cullin immunoisolation. Accession numbers for interacting proteins identified that could be classified into RING, SKP or client adaptor classes are given. Full details of LC-MSMS data are available in the supplementary data. Adaptors are classified based on the presence of a specific identifiable domain or architecture. 1; No transfectants obtained. 2; No adaptors identified. 3: No RING-domain ligase identified. The clade assignment from Fig. 1 is shown at left while the likely *H. sapiens* ortholog is shown from TbCul-A and -D.

Clade	Hs	Tb	Cullin	Rbx	Skp-1	Client adaptors
Culα	CUL1	TbCul-A	Tb927.8.5970	TbRbx1 Tb927.10.1810	TbSkp-1 Tb927.11.6130	F-box: Tb927.8.1380; Tb927.5.700; Tb927.7.4300; Tb927.10.12060; Tb927.10.410; Tb927.4.3000
Culκ		TbCul-B^1^	Tb927.10.7490			
Culκ		TbCul-C^2^	Tb927.8.5210			
Culκ	CUL4a	TbCul-D	Tb927.3.1290	TbRbx1 Tb927.10.1810		DDB1: Tb927.6.5110; DCAF Tb927.11.3190; Tb927.8.840
Culκ		TbCul-E	Tb927.11.11430	TbRbx1 Tb927.10.1810	TbSkp-1.2 Tb927.9.8370	Kelch: Tb927.8.6140; Tb927.11.11450;Tb927.5.1980; Tb927.1.820
Culκ		TbCul-F	Tb927.10.6930	TbRbx1 Tb927.10.1810	TbSkp-1.4 Tb927.10.14310	
Culκ		TbCul-G^3^	Tb927.4.4760		TbSkp-1.3 Tb927.11.13330	LRR: Tb927.4.3100; Tb927.6.1490; Tb927.10.10190

**Fig. 1:**
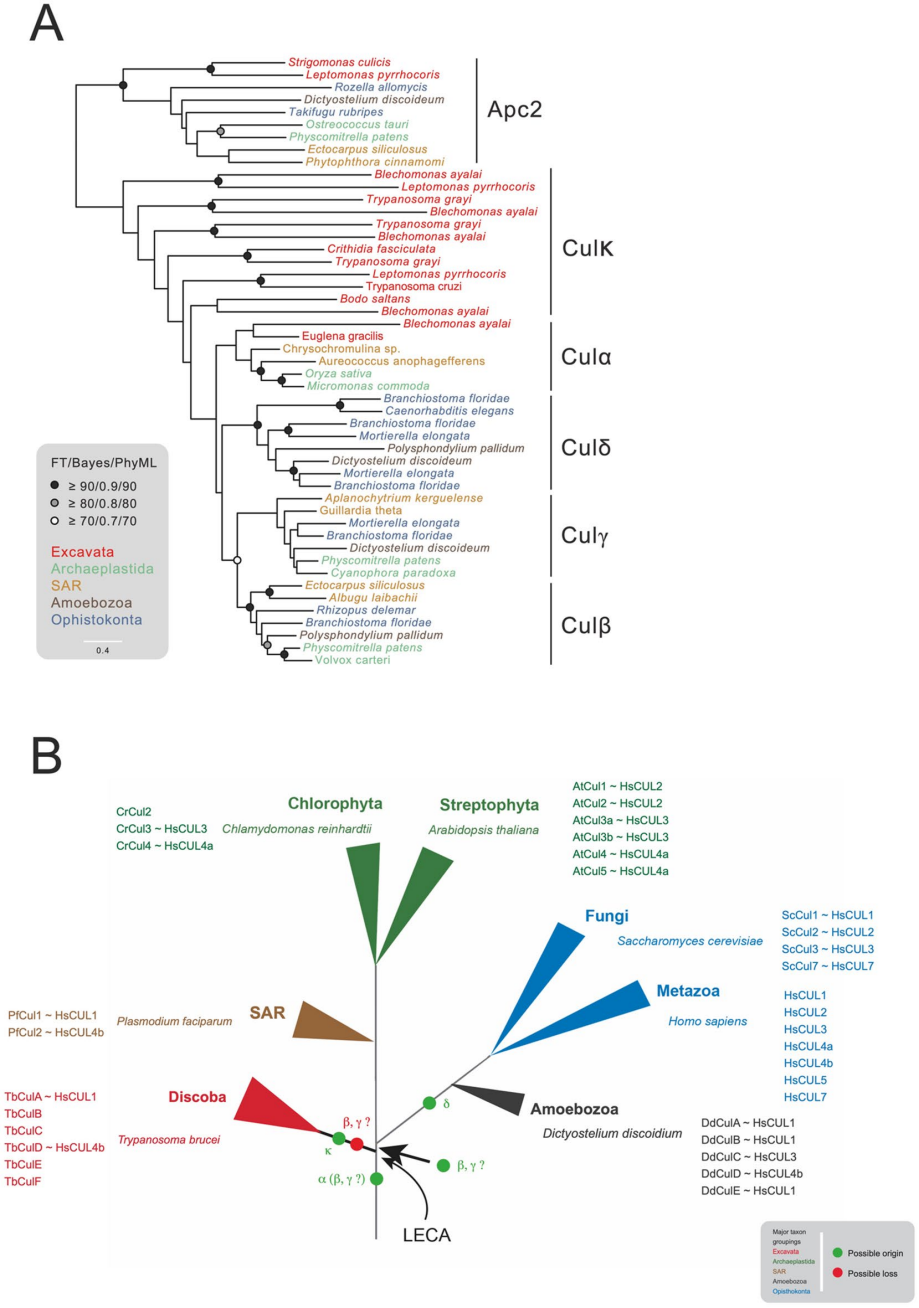
Four ancient clades and the expansion of cullins in Excavata. (**A**) Reconstruction of cullin phylogeny with representation from every eukaryotic supergroup reflects the proposed notion of three ancient clades (Cukα, Culβ and Culγ). An additional amorphea clade was identified (Culδ) which likely arose post-LECA. Only one of the seven cullins from Excavata, Cul-A, is part of the major clades and the remaining, appear to be kinetoplastid specific as Culκ (κ = kinetoplastida). Taxa are colour-coded based on supergroup, indicated on the bottom right. The anaphase-promoting complex subunit 2 (Apc2) was used as an outgroup. Support values are shown when > 70% in each of the three inference methods of phylogeny. The scale bar indicates amino acid substitution rate. (**B**) Cullin nomenclature and orthology across eukaryotes. The cullin names and *H. sapiens* orthologs are given beside important model organisms/pathogens. Positions of origins and possible losses of cullin clades are indicated in neon green and British postbox red respectively. Taxa are colour-coded as in (**A**).

Besides the APC2 outgroup, the phylogeny possesses five major clades, formed by clusters containing orthologs of CUL-3 and CUL-4a, consistent with earlier analysis and designation as clades Culα, Culβ and Culγ^40^ (Fig. 1). CUL-1 and multiple animal, fungal and amoebozoan orthologs did not cluster with Culα but formed a fourth clade, which we term Culδ. All cullins from Archaplastida (plants) and SAR (stramenopiles, alveolates and rhizarians) cluster into Culα, Culβ or Culγ, indicating that these ancestral clades span eukaryotic supergroups beyond the Archaeplastida and Opisthokonta and hence are more ancient than previously suggested^40^. The branching order suggests that Culα likely diverged first, followed by Culβ and Culγ, while Culδ is taxon-restricted and hence likely arose after the last eukaryotic common ancestor (LECA).

Of cullins in the Excavata lineage, including trypanosomes, Cul-A clustered within the Culα clade, represented here by sequences from *Euglena gracilis* and *Blechomonas ayali*. However, the remaining paralogs, Cul-B to Cul-G, were excluded from Culα, Culβ, Culγ or Culδ, indicating they are either lineage-specific or so divergent that they cannot be placed with confidence within the pan-eukaryotic clades. Long branch lengths suggest phylogenetic artefact, but additional reconstructions calculated by removing subsets of sequences (not shown), continued to support this topology (but see below). We designate this cluster as Culκ, and suggest that many cullins in trypanosoma, and probably other excavates, may have lineage-specific origins, and hence possible functional divergence (*albeit* that we cannot exclude extreme divergence compromising the phylogenetic topology). With the exceptions of Cullin-1 (or A) and Cullin-4a (or D) where the client adaptor subunit families are congruent with opisthokont cullins, we suggest naming trypanosome cullins with letters as nomenclature using numbers implies orthology, which is not fully supported. We note similar nomenclature issues with cullin cohorts in other organisms such as *Dictyostelium discoidium* (Fig. 1).

Considering cullin evolution in the context of the eukaryotic tree of life highlights several features (Fig. 1). Firstly, while Culα was likely present pre-LECA, because of an inability to fully resolve excavate cullins it remains unclear if Culβ and Culγ arose prior to the LECA, and were specifically lost in excavates, or arose after speciation of the Excavata from the main eukaryotic lineage. Furthermore, we find that orthologs of *H. sapiens* CUL1 and CUL4a and CUL4b are commonly retained in model organisms. There are also several examples of likely lineage-specific expansions, e.g. *D. discoidium* CulA and CulB are both probable HsCUL1 orthologs, while in *A. thaliana* Cul1 and Cul2 are orthologs of HsCUL2, and *A. thaliana* Cul4 and Cul5 are both orthologs of HsCUL4a^37,38,41^.

### Cullins are associated with lineage-specific and conserved subunits

In higher eukaryotes complexes from each primordial cullin clade, Culα, Culβ and Culγ, recruit a specific family of client recognition adaptors, while Culδ complexes possess novel adaptors, including VHL and SOC proteins^35,40^. Only one cullin from *T. brucei* has been studied to date and designated as TbCul-4a; this complex possesses client adaptors orthologous to DDB1 and DCAF^19^ (Table 1), while a likely CUL-1 ortholog possessing F-box client adaptors has been characterised in *L. infantum*^33^. In the main, transcriptome data indicate that TbCul-A, B, C, E and F, together with TbRbx-1, are upregulated in procyclic culture forms, while TbCul-D and G appear constitutive (https://tritrypdb.org/tritrypdb/app). Understanding the composition of the cullin complexes in trypanosomes was chosen in part to address concerns over cullin phylogenetic reconstruction as well as to explore the potential for novel adaptors not found in canonical organisms.

To address this we used immunoisolation/mass spectrometry to determine a composition for each trypanosome cullin complex. Six of seven cullins (TbCul-A, TbCul-C, TbCul-D TbCul-E, TbCul-F and TbCul-G) were endogenously tagged at the C-terminus with three HA epitopes and expression confirmed by Western blotting (Figure S1), with TbCul-C and TbCul-F present at significantly lower abundance compared with the other cullins. We did not observe significant impact on proliferation of the tagged strains. Multiple attempts to tag TbCul-B were unsuccessful, and this gene was also refractory in high throughput efforts, and with a different tag^42,43^.

Given the dynamic nature of client adaptor/cullin interactions we used cryo-milling for rapid disruption of cells under near native conditions, a method for one step capture of complexes and validated by multiple studies^44,45^. Multiple buffer conditions were evaluated for each isolation and captured proteins visualised using silver-stain SDS-PAGE, allowing identification of appropriate biochemical conditions to optimise signal to noise ratios (Figure S1). Immunocomplexes were analysed using liquid chromatography tandem mass spectrometry (LC-MSMS) and compared to an immunoisolation from untagged parental cells under identical conditions (Supplementary Tables S2 and S3).

**Fig. 2:**
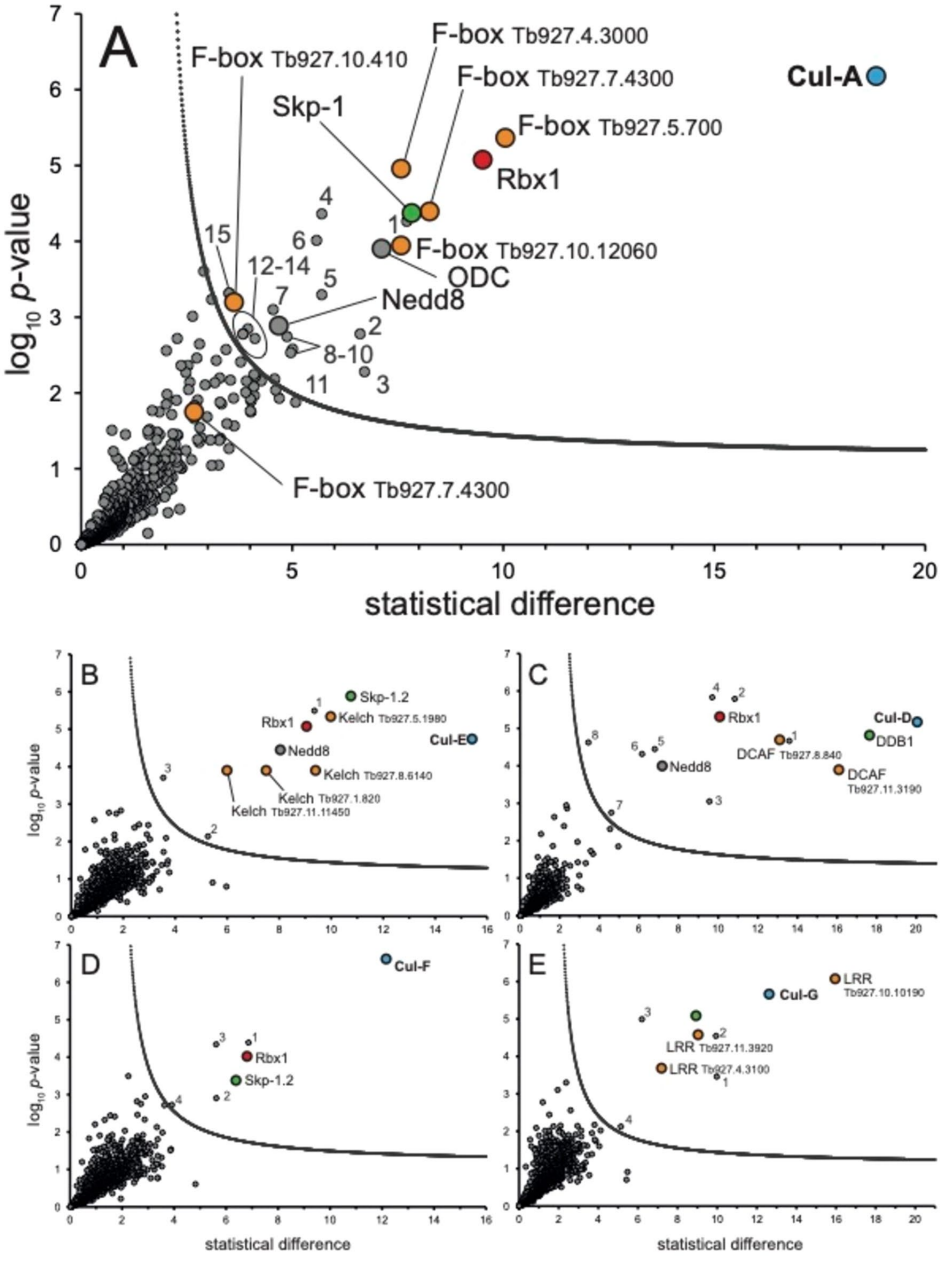
Proteins co-eluting with TbCul immunoprecipitations. Proteins coeluted with the HA-tagged TbCullins after immunoprecipitation using magnetic anti-HA nanobeads and identified using mass spectrometry. The statistical *t*-test difference of LFQ intensities for the tagged cell line versus the parental is plotted against the -log10 transformed p-values (n > 3). Cutoff curves were calculated for an FDR of 5% and minimum fold change S0 at 1.0. Potential Cullin-RING complex components are colour coded based on their expected role (compare Fig. 1). (**A**) TbCul-A immunoprecipitation. ODC (ornithine decarboxylase; Tb927.11.13730) and Nedd8 (ubiquitin-like modifier; Tb927.4.2540) are highlighted and the remaining proteins within the statistical significance cutoff are labeled by numbers: 1 = Tb927.10.11830; 2 = Tb927.9.6660; 3 = Tb927.5.440, trans-sialidase; 4 = Tb927.11.13740, GRESAG (gene related to expression site-associated genes); 5 = Tb927.11.1480, GRESAG; 6 = Tb927.11.10370, glycosyl hydrolase-like protein; 7 = Tb927.4.5240, glycosyltransferase; 8 = Tb927.7.7470, GRESAG; 9 = Tb927.11.13130, membrane-bound acid phosphatase 1; 10 = Tb927.9.2260; 11 = Tb927.10.6780, vacuolar protein sorting-associated protein 45 (VPS45); 12 = Tb11.v5.1016, GRESAG; 13 = Tb927.11.3350, phospholipid-transporting ATPase 1; 14 = Tb927.10.12290, glycosyltransferase; 15 = Tb927.9.11480. (**B**) TbCul-E immunoprecipitation. Nedd8 (Tb927.4.2540) is highlighted and the remaining proteins within the statistical significance cutoff are labeled by numbers: 1 = Tb927.6.4360; 2 = Tb927.8.5580, VPS13; 3 = Tb927.11.9550, replication factor C subunit. (**C**) TbCul-D immunoprecipitation. Nedd8 (Tb927.4.2540) is highlighted and the remaining proteins within the statistical significance cutoff are labeled by numbers: 1 = Tb927.11.1080, NOP60; 2 = Tb927.6.650; 3 = Tb927.11.8960; 4 = Tb927.8.1230; 5 = Tb927.8.760, NOPP44/46–1; 6 = Tb927.6.2640, Kap60; 7 = Tb927.11.4280; 8 = Tb927.8.750, nucleolar RNA-binding protein. (**D**) TbCul-F immunoprecipitation. Proteins within the statistical significance cutoff are labeled by numbers: 1 = Tb927.11.9840; 2 = Tb927.10.9090, DCNL-like; 3 = Tb927.8.6510, UBC12; 4 = Tb927.10.2020, Hexokinase. (**E**) TbCul-G immunoprecipitation. Proteins within the statistical significance cutoff are labeled by numbers: 1 = Tb927.10.7290, flagellar member 2; 2 = Tb927.4.3620, protein phosphatase 1; 3 = Tb927.4.3640, protein phosphatase 1; 4 = Tb927.8.5580, VPS13. For complete, detailed annotation see Supplementary Table S2.

In isolations using TbCul-A and TbCul-D as bait we identified multiple F-box and DCAF/DDB1 paralogs respectively amongst the enriched proteins (Fig. 2), together with an ortholog of Skp-1 and Rbx-1 (Table 1). The protein–protein interactions of TbCul-D are consistent with previous reports^19^, while TbCul-A interaction partners, specifically F-box proteins, are consistent with inclusion within the Culα clade. The presence of DCAF adaptors associated with TbCul-D is also consistent with this complex being homologous to Cul-4a. By contrast, apart from Skp-1 paralogs, analysis of other trypanosome cullins failed to identify canonical substrate recognition subunits defining Culα, Culβ or Culγ clades.

### Conserved compositions in trypanosome cullin protein–protein interactions

Two of six trypanosome cullin complexes demonstrated homologous composition with higher eukaryotes. Five F-box proteins were significantly enriched in TbCul-A immunoisolations, alongside orthologs of Rbx-1 and Skp-1, which in *H. sapiens* comprises the archetypal SCF complex. We also identified Nedd8, two glycosyltransferases, a glycosylhydrolase, four GRESAG4 proteins, ornithine decarboxylase (ODC), a phospholipid-transporting ATPase, a kinase, a phosphatase, a *trans*-sialidase, ESAG9 and two hypothetical proteins (Fig. 2A). While the extent to which these additional proteins form a complex with trypanosome SCF is unclear, Nedd8, an ubiquitin-like modifier, has been identified in *T. brucei* cullins^38^, and suggests that Nedd8 controls activation of trypanosome cullin activity through conformational change upon conjugation, as in higher eukaryotes^6^. ODC, a key metabolic enzyme in the essential polyamine biosynthetic pathway, is the target of the human African trypanosomiasis frontline drug eflornithine that covalently binds to the ODC active site. Of the remaining identified proteins, *trans*-sialidases, protein products of genes related to expression-site associated genes (GRESAGs) and expression-site associated genes (ESAGs) are all products of large paralog gene families and frequently identified in proteomics in *T. brucei*. Overall, the composition of the TbCul-A/Cul-1 complex is consistent with both our phylogeny identifying membership to the Culα clade and evidence from *L. infantum*^33^, which suggests a high level of conservation for this complex.

Among the most abundant proteins associated with TbCul-D were two proteins with DCAF domains and corresponding adaptors DDB1 and Rbx-1 (Fig. 2). Further, Nedd8 was detected, together with five hypothetical proteins, nucleolar proteins 44/46 and 60, a nucleolar RNA-binding protein and importin-α. The majority of TbCul-D interactors have evidence for nuclear localisation, including TbCul-D itself, based on high throughput tagging^44^, and the presence of a nuclear localization signal (NLS). For example, hypothetical proteins Tb927.8.1230 and Tb927.6.650 possess a nucleoplasmin-like fold and localise to the nucleolus or a DNA damage-binding protein component respectively. *H. sapiens* CUL4 includes RBX1, DDB1 (damaged DNA binding protein-1) and DCAF domain proteins and has roles in DNA damage repair, is located in the cell nucleus and interacts with other nuclear proteins. Hence, HsCUL4a and TbCul-D occupy a common nuclear location as well as have similar compositions, suggesting these complexes are homologs, despite the failure of TbCul-D to be included in an ancient phylogenetic clade.

### Evidence for lineage-specific cullin subunits

Immunoisolations of TbCul-E, TbCul-F and TbCul-G did not identify F-box, DCAF or BTB proteins, i.e. canonical client adaptor proteins, but did contain novel Skp-1 paralogs and protein families with domains characteristic of client receptors, albeit with distinct architectures. This supports the hypothesis that TbCul-E, TbCul-F and TbCul-G are lineage-specific complexes (Fig. 2; Table 1). The largest group of proteins associated with TbCul-E are four proteins possessing three to six kelch domains. These domains span a ~ 50 amino acid β-propeller structure frequently involved in protein–protein interactions and also present in proteins bearing F-box and BTB domains, although F-box and BTB domains are absent from TbCul-E interacting proteins. Significantly, several kelch domain-containing proteins including KLHDC2, KLHDC3, and KLHDC10 interact with the mammalian Cullin2-RING (CRL2) complex^46,47^. Other interactors of TbCul-E include a Skp-1 paralog, Skp1.2. Furthermore, Nedd8, replication factor C subunit four and vacuolar protein sorting protein 13 (Vps13) were detected (Fig. 2); excepting Nedd8 and Skp-1.2, the physiological relevance of these protein–protein interactions is unclear.

For TbCul-F we identified interactors including TbRbx1 and a further Skp-1 paralog, designated TbSkp-1.4 (Tb927.10.14310). TbCul-F is the sole *T. brucei* cullin for which there is no direct evidence for neddylation^38^, but as E2 and E3 neddylation cascade enzymes were identified, specifically TbUbc12 and a DCNL-like protein respectively, it is probable that TbCul-F is neddylated. Another TbCul-F interaction partner is hexokinase; significantly in *H. sapiens* hexokinase is ubiquitinated by Perkin E3 ligase, but as trypanosomes lack a Perkin ortholog, ubiquitylation of trypanosome hexokinase cannot use this mechanism and hence regulation may be mediated via TbCul-F.

TbCul-G interacts with a further Skp-1 paralog, TbSkp-1.3 (Tb927.11.13330) (Fig. 2; Table 1), and three leucine-rich repeat proteins (LRR). LRR domains are frequently involved in protein–protein interactions and present in some F-box proteins in metazoa as the FBXL family^4^, making it highly likely that these LRR proteins are client recognition subunits. Further, three hypothetical proteins are also enriched, Tb924.4.3620, Tb927.4.3640 and Tb927.10.7290. The former two are phosphatases and a tandem duplication with near identical sequence. Tb924.4.3620 is clearly localised to the flagellum by high throughput analysis^42,43^. The third protein, Tb927.10.7290 is FLAM2, a component of the flagellum/PFR assembly^48^. As this cohort of proteins are concordant, we suggest that TbCul-G possesses novel LRR-domain client recognition subunits with roles in modulating flagellar function and/or assembly; significantly these resemble FBXL adaptors but lack the F-box itself.

### Diversity of cullin client adaptor repertoire

Some lineage-specific cullin complexes have evolved client adaptors beyond canonical proteins, for example the animal-specific Cul2 and Cul5 belong to the Culα clade but recruit EloB and EloC as adaptors. In organisms lacking Cul2 and Cul5, EloB and EloC regulate tRNA polymerase II, presumably an example of repurposing^49^. EloB has a Skp-1-like domain similar to the Skp-1 paralog proteins identified in immunoisolates from TbCul-E, TbCul-F and TbCul-G. To investigate the origins of these proteins we constructed a phylogeny across eukaryotes, using EloB as outgroup (Figure S2). TbSkp-1 belongs to a clade with members from every supergroup, including *bona fide* SKP-1 orthologs in all sampled lineages. Significantly, TbSkp-1.2, TbSkp-1.3 and TbSkp-1.4 orthologs are restricted to excavates; TbSkp-1.2 is only found in the Euglenozoa while TbSkp-1.3 and TbSkp-1.4 are trypanosomatid specific, indicating lineage-specific expansions.

**Fig. 3:**
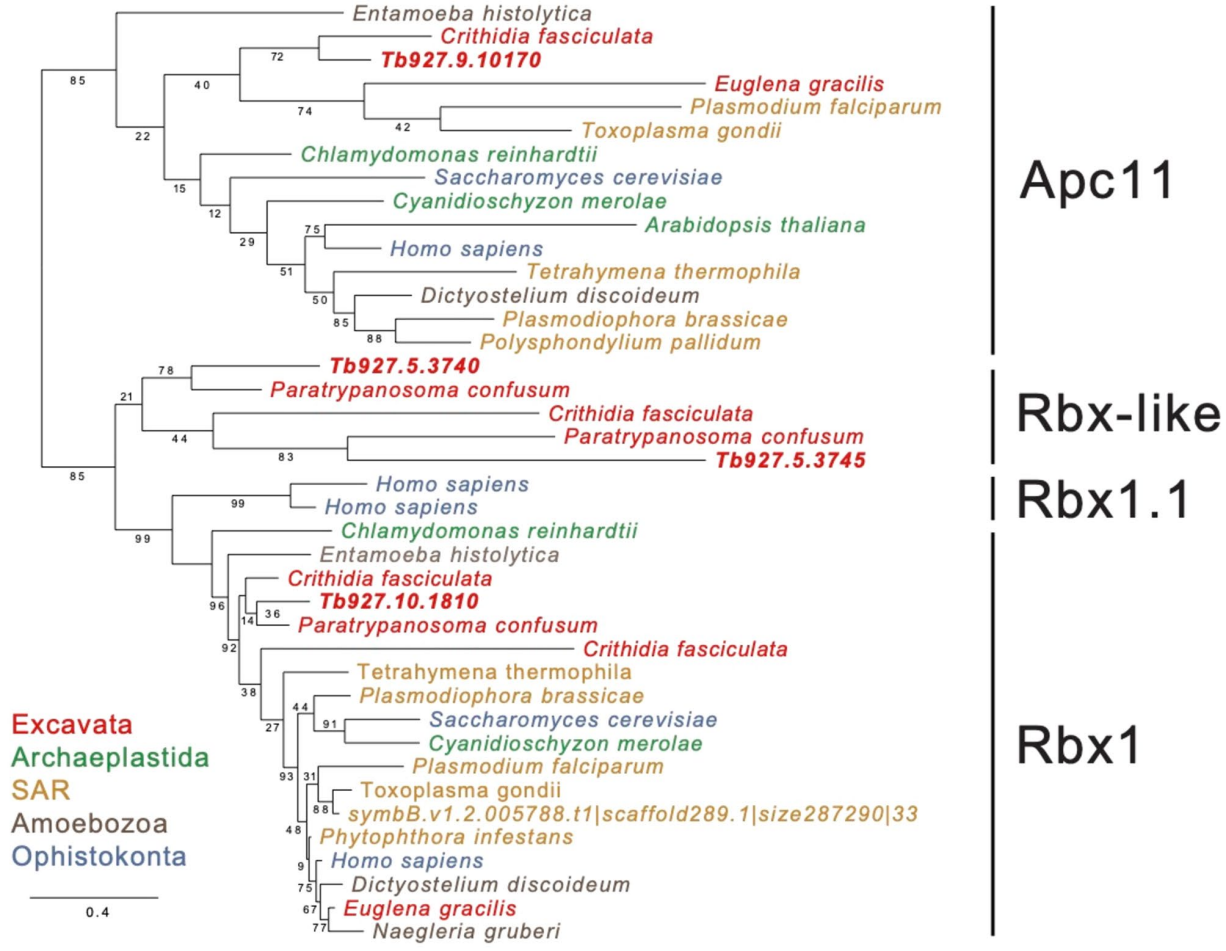
RBX-like proteins in Excavata. Maximum likelihood phylogenetic tree of RBX proteins in Eukaryota indicates a unique expansion in Kinetoplastida, that have an RBX1 ortholog and two novel RBX-like proteins. The clades identified are labelled with black bars and each taxon is coloured based on supergroup affiliation. The accession numbers for *T. brucei* sequences are given whereas all other sequences are annotated by species. The anaphase-promoting complex subunit 11 (Apc11) was used as outgroup. Statistical support is indicated for PhyML, and the scale bar indicates amino acid substitution rate.

Given this, we asked if the Rbx family has undergone a similar evolutionary pathway. Orthology searches spanning eighteen species representing all supergroups were performed using Rbx-1 and Rbx-2 orthologs with APC subunit 11 as outgroup (Fig. 3). This resulted in the identification of three Rbx-like proteins in excavates; only the Rbx-1 ortholog was identified in TbCul-A, -D, -E and -F immunoisolations. The two apparent cullin-independent Rbx-like paralogs, Tb927.5.3740, which is glycosomal and Tb927.5.3745, which is cytoplasmic, are a trypanosome-specific expansion (Figure S6); while Tb927.5.3745 is essential^31,42,43^ both are of unknown function and were not identified in any cullin immunoisolation.

### Excavata-specific client recognition subunits

There are over 70 F-box proteins in *H. sapiens,* each potentially able to recruit proteins for ubiquitylation by CUL1^50^. For complexes formed by CUL4a, there are nearly 90 human DCAF proteins^51^. We sought to determine if there was conservation of F-box and DCAF proteins associated with TbCul-A and TbCul-D and Cul-1 and Cul-4a in metazoa.

F-box protein sequences from *H. sapiens* and *A. thaliana* failed to identify a *bona fide T. brucei* F-box protein by BLAST, but using “F-box” as a text search at TriTrypDB^52^ and confirmation using HMMER, InterPro and SMART we identified 16 proteins, which included all F-box proteins identified by immunoisolation of TbCul-A. These 16 F-box protein sequences were used as queries in a BLASTp search against a selection of Kinetoplastida (Figure S3). Of these F-box proteins, one is restricted to African trypanosomes and close relatives such as *T. grayi* (Tb927.10.12060) and five have orthologs in *T. cruzi* (Tb927.7.4300; Tb927.8.1380; Tb927.5.700; Tb927.10.410; Tb927.4.3000) of which only two are also present in *Leishmania* (Tb927.10.410; Tb927.4.3000). This suggests lineage-specific evolution of the F-box repertoire in kinetoplastids.

Searches using 27 *H. sapiens* DCAF protein sequences retrieved three hits from *T. brucei* (Tb927.8.4210, Tb927.9.11250 and Tb927.9.9090), orthologs of HsWDR15b, HsDCAF13 and HsDCAF7 respectively. These, in addition to the DCAF proteins identified following immunoprecipitation of TbCul-D (Tb927.11.3190 and Tb927.8.840), were used as queries to reconstruct a phylogeny for this family of substrate adaptors in Kinetoplastida (Figure S4). Every DCAF identified in *T. brucei* has orthologs in *T. cruzi* and *Leishmania* spp. except Tb927.8.840, which is restricted to *Trypanosoma*. Furthermore, we also investigated representation of Kelch domain proteins across kinetoplastids, identifying four clades with the presence of at least two expansions in the trypanosomes (Figure S5). Overall these data reveal plasticity within the client adaptor family in kinetoplastids and by inference provides evidence for significant lineage-specific evolution of these cullin complex components.

### Knockdown of selected trypanosome cullins identifies specific functions

To investigate trypanosome cullin functions we selected TbCul-A and TbCul-E. TbCul-A is a conserved cullin complex and in trypanosomes interacts with ODC, the target of the suicide-inhibitor eflornithine, which remains in use for treatment of trypanosomiasis^53^, while TbCul-E was chosen based on essentiality and possession of novel client adaptors^54^. Previous studies focusing on kinetoplastid cullin complex components have targeted Skp-1 or specific client adaptor proteins^18,19^, which, in the former, is likely to impact multiple complexes and neither study investigated the impact on the cellular proteome. Cell lines were created for inducible knockdown and quantitative reverse transcription PCR (qRT-PCR) used to assess cullin transcript levels after induction of RNAi, with an over 75% decrease in both cases (Figure S7). LC-MSMS was used to quantify the effects of TbCul-A and TbCul-E silencing on protein abundance.

**Fig. 4:**
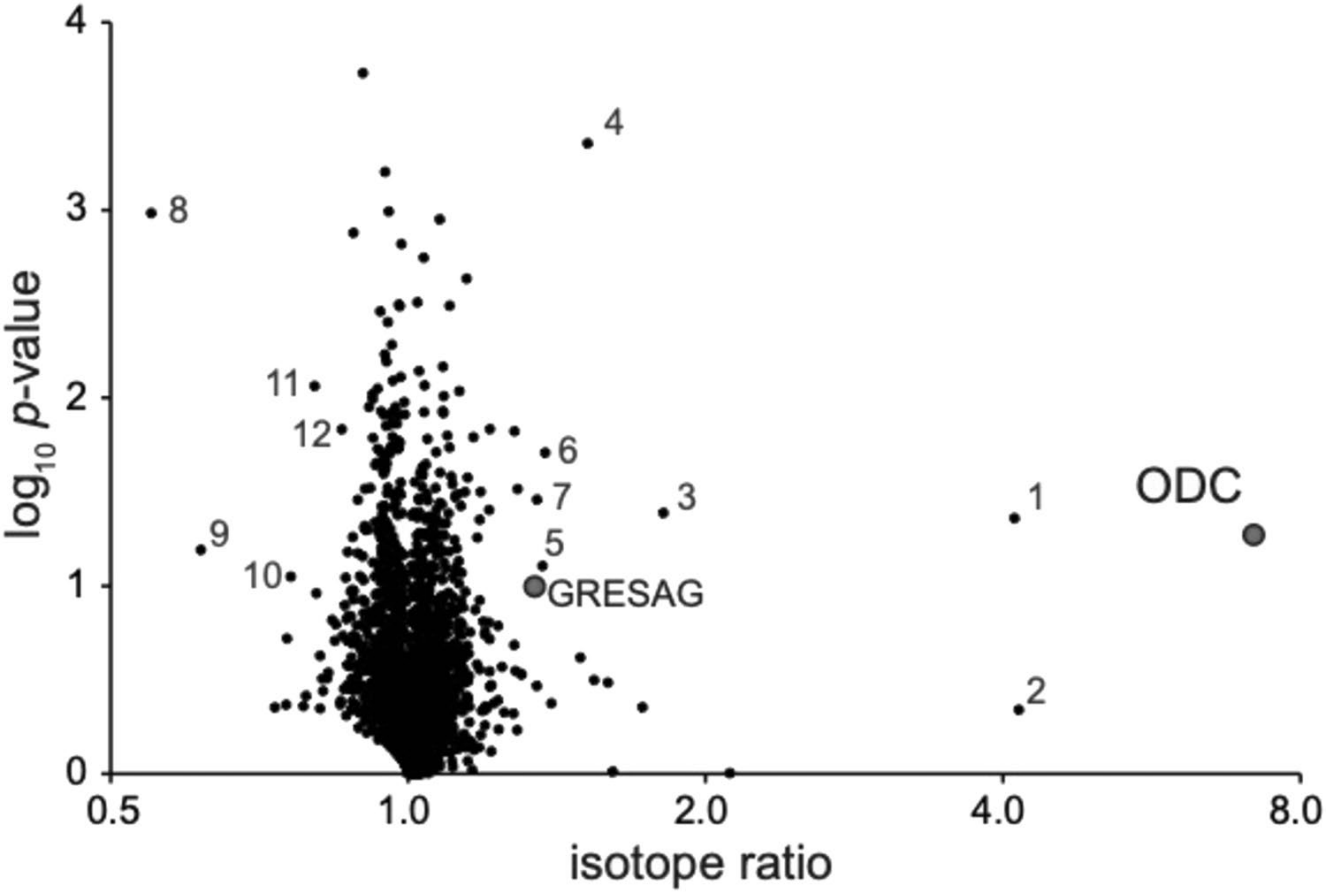
Knockdown of TbCul-A increases TbODC abundance. Landscape of *T. brucei* proteome changes 24 h after silencing of TbCul-A. The volcano plot shows SILAC ratios between induced and uninduced cells (each n = 3) plotted against –log10 transformed p-values. Proteins identified in TbCul-A immunoisolates are labelled and respective datapoints highlighted in red (GRESAG (gene related to expression site-associated genes), Tb927.7.7470; ODC (ornithine decarboxylase), Tb927.11.13730). Selected additional proteins are labelled by numbers: 1 = Tb927.7.830, telomerase-associated protein; 2 = Tb927.4.4410, GRESAG; 3 = Tb927.4.4440, GRESAG; 4 = Tb927.5.2090, putative kinesin; 5 = Tb927.10.11660; 6 = Tb927.6.4460/Tb927.6.4410, S-adenosyl decarboxylase; 7 = Tb927.7.6070, GRESAG; 8 = Tb927.4.2920; 9 = Tb927.11.7520; 10 = Tb927.11.2230, carnitine O-acetyltransferase; 11 = Tb927.6.1850, quiescin sulfhydryl oxidase; 12 = Tb927.8.4890, endoplasmic reticulum oxidoreductin. For complete, detailed annotation see Supplementary Table S5. Significantly, none of the proteins impacted by the knockdown, with the exception of TbODC and one GRESAG, were identified in the immunoprecipitation of TbCul-A.

TbCul-A silencing had no detectable impact on proliferation, as reported previously^18^. However, we did find decreased choline/carnitine o-acetyltransferase (CRAT), ER oxidoreductin 1 (ERO1), quiescin sulfhydryl oxidase (QSOX) and two lineage-specific likely axoneme-associated hypothetical proteins (Tb927.4.2920 and Tb927.11.7520) (Fig. 4, Supplementary tables 5 and 7). In animals, fungi and vascular plants, QSOX and ERO1 are components of the ER oxidative protein folding system, facilitating disulphide bond formation^55^. CRAT is involved in fatty acid biosynthesis and regulates levels of acyl-CoA and CoA^56^. Considering that none of these proteins were identified in the immunoprecipitation of TbCul-A their decreased abundance following RNAi is unclear.

By contrast, there was concordance between proteins with increased abundance and TbCul-A interactors upon silencing, suggesting these proteins are *bona fide* TbCul-A clients. Most prominent were ODC and GRESAG paralogs, both of which were identified in TbCul-A isolations. Also impacted were S-adenosyl decarboxylase which, together with ODC, is a component of the polyamine biosynthesis pathway. Two kinesins, two hypothetical proteins restricted to the kinetoplastida, Tb927.11.10380 (which contains a RNI-domain that is associated with F-box domains in some proteins), and Tb927.10.11660, a nuclear protein with a predicted VSG-related helical bundle architecture and nucleolar protein 61 (NOP61) were also increased in abundance.

**Fig. 5:**
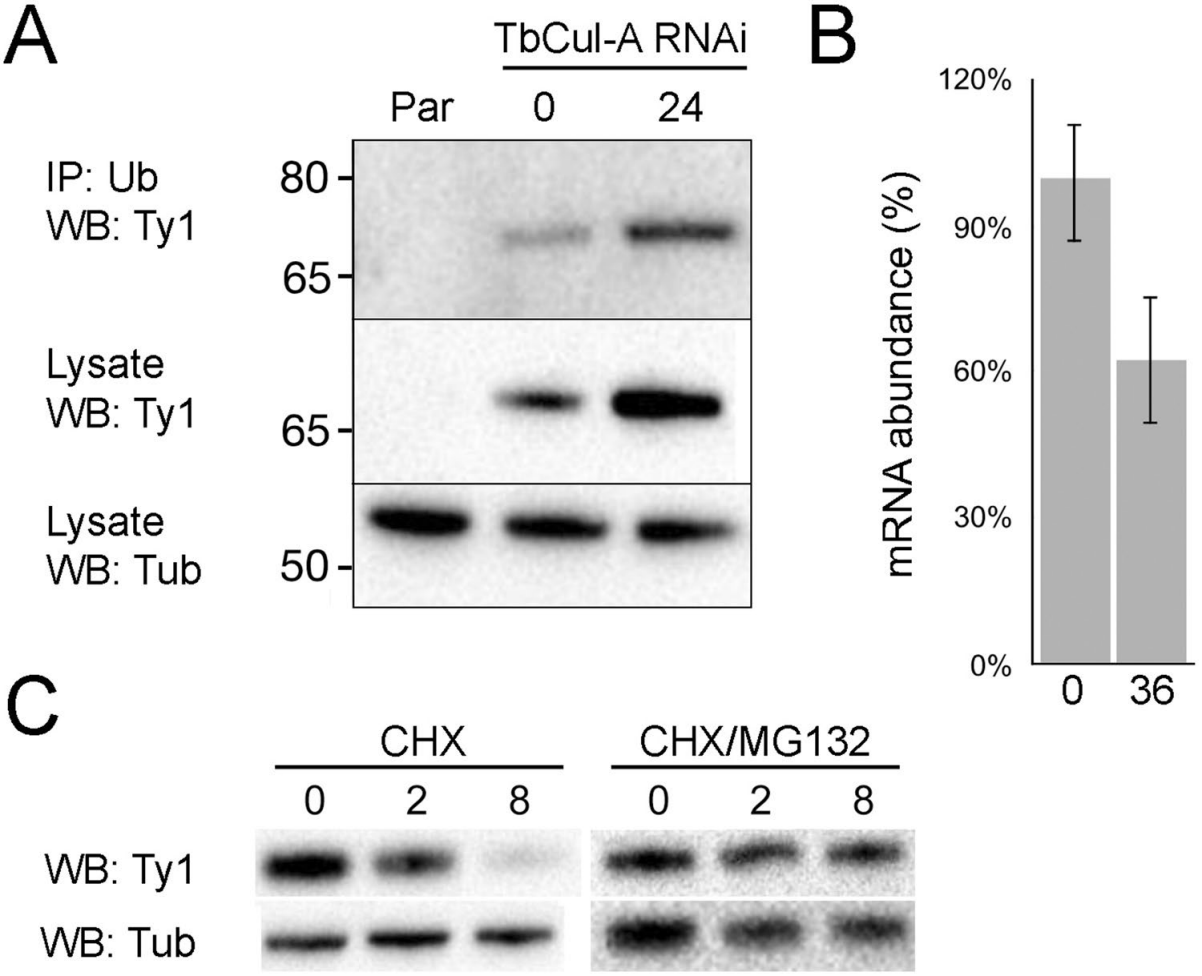
TbODC turnover is mediated by ubiquitylation and proteasome activity. (**A**) Elution of TbODC from ubiquitin-recognising beads. TbODC with a Ty1-tag endogenously fused to its C-terminus was captured by UbiQapture-Q beads after incubation with cell lysate. Par; parental line, antibody against tubulin was used as a loading control. The intensity of TbODC-10xTy1 detected from immunoprecipitation and from the total fraction increased following induction of TbCul-A RNAi for 24 h with tetracycline while quantitative RT-PCR against TbODC (**B**) indicates that levels of TbODC mRNA are stable following RNAi after 36 h. Black lines indicate sub-panels derived from different blots. Panel C: Degradation of TbODC is mediated by the proteasome. Left; turnover of TbODC as measured following inhibition of protein synthesis with cyclohexamide (CHX) and right; turnover of TbODC as measured following inhibition of protein synthesis and proteasome activity. Blot is representative of four replicates. Numbers above/below lanes indicate time post RNAi induction in hours. CHX; cyclohexamide and antibody against tubulin was used as a loading control. Gaps between sub-panels indicate images derived from different blots.

To establish ODC as a TbCul-A client protein, the ODC gene was endogenously tagged with ten Ty1 epitopes in the TbCul-A RNAi cell line. To determine if TbODC is ubiquitylated we used affinity isolation from whole cell lysates of cells with and without TbCul-A silenced and probed with anti-Ty1 antibody (Fig. 5). ODC::10xTy was detected in eluates from ubiquitin-recognising beads both before and after knockdown of TbCul-A as a single band at ~ 70 kDa, consistent with ubiquitylation. We did not detect polyubiquitylated forms, but the half-life of these forms is extremely low. Moreover, the Western blot signal was more intense in TbCul-A silenced cells, while RT-qPCR indicated that TbODC mRNA levels were not increased following knockdown. Further, turnover of ODC is MG132-sensitive, and which is near complete after eight hours in control cells but strongly protected by the proteasome inhibitor, indicating a proteasome-dependant mechanism, and consistent with ODC as a TbCul-A ubiquitin ligase substrate.

**Fig. 6.**
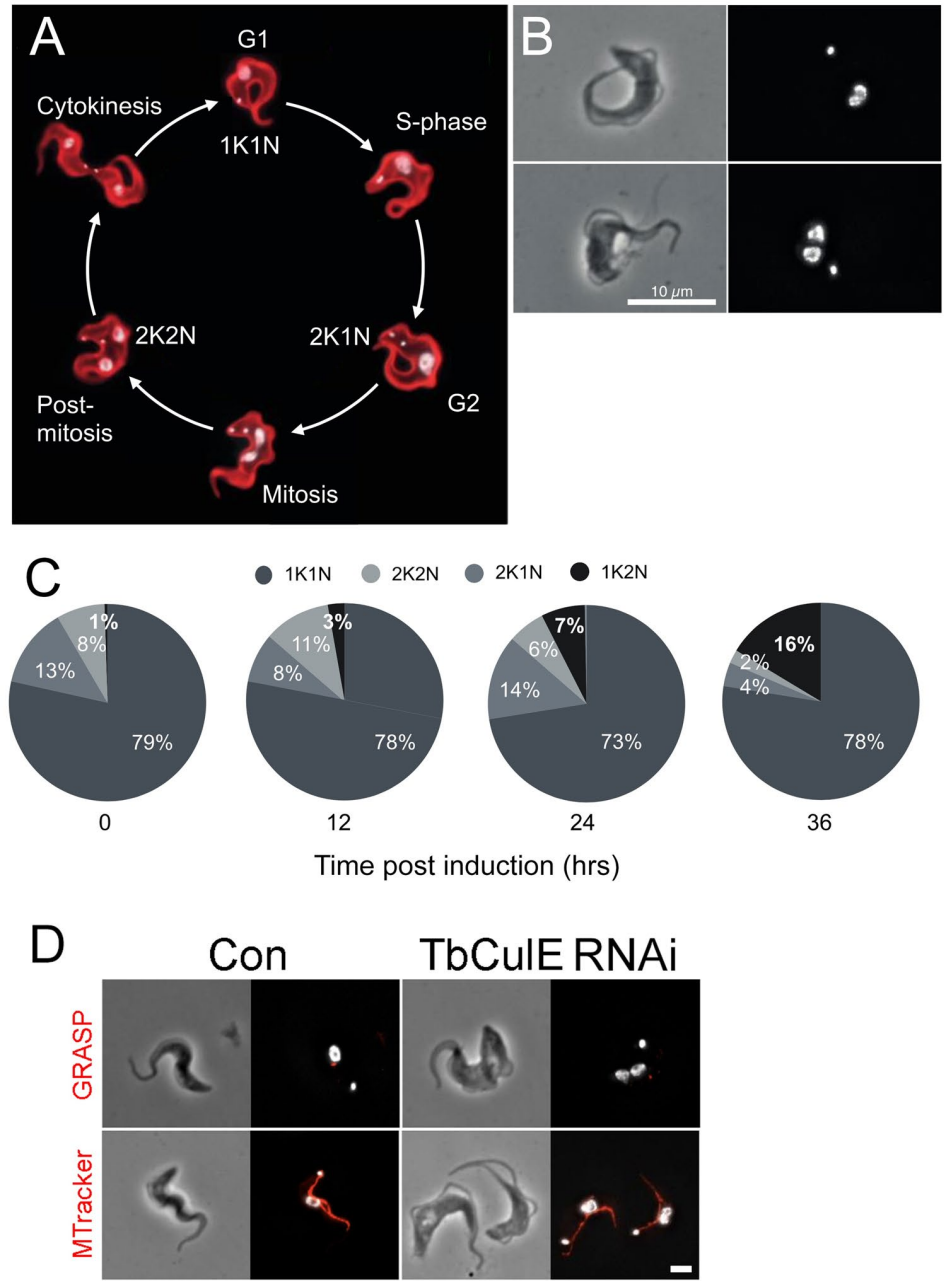
1K2N phenotype produced by knockdown of TbCul-E. (**A**) Cell cycle of bloodstream form *T. brucei* and the corresponding number of kinetoplasts and nuclei in each stage (adapted from Glover et al., 2019). (**B**) Example of DAPI stained cells with one kinetoplast and two nuclei (1K2N) observed after induction of TbCUL-E RNAi. (**C**) karyotype progression throughout the knockdown (number of cells counted > 300 at each time). Note totals in some plots exceed 100% due to rounding. Panel D; Immunofluorescence of TbGRASP and mitotracker, indicating that in 1K2N cells the Golgi complex also fails to replicate. DNA stained by DAPI is in white, TbGRASP and mitotracker in red and scale bar is 2 μm.

Knockdown of TbCul-E is lethal (Figure S8), with emergence of cells possessing one kinetoplast and two nuclei (1K2N) following silencing (Fig. 6), broadly consistent with an earlier report^19^. Replication of the kinetoplast is concurrent with development of a second flagellum, one of the earliest events indicating progression from G_1_ into cytokinesis and preceding nuclear division. A 1K2N karyotype is not a canonical cell cycle form and suggests aberrant nuclear division prior to kinetoplast/basal body replication. Given the absence of detectable 1K0N cytoplasts, it is unlikely that these 1K2N cells arise from defective cytokinesis and more likely from disrupting coordination between replicating nuclei and kinetoplasts, bypassing the normal mitotic checkpoints.

**Fig. 7:**
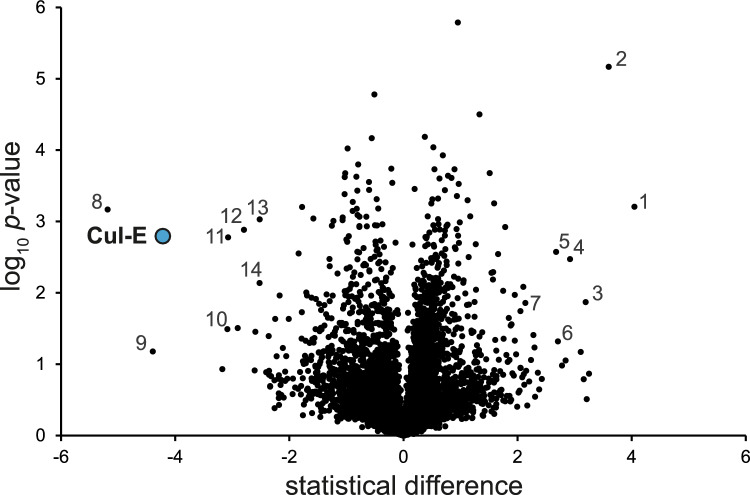
Impact of TbCul-E knockdown on global proteome. Landscape of *T. brucei* proteome changes 24 h after silencing of TbCul-E. The volcano plot shows the statistical difference between induced and uninduced cells (each n = 3), calculated from LFQ intensities, and plotted against -log10 transformed p-values. TbCul-E is highlighted in blue and labelled. Selected additional protein groups are labelled by numbers: 1 = Tb927.11.17010, rab5-interacting protein; 2 = Tb927.1.4900, ESAG11; 3 = Tb927.1.4900, VSG-related; 4 = Tb927.11.7770, oxidoreductase-like; 5 = Tb927.5.2120; 6 = Tb927.4.710; 7 = Tb927.8.1150, kinetochore protein 9; 8 = Tb927.10.8810; 9 = Tb927.11.7520; 10 = Tb927.7.3250, ESAG6; 11 = Tb927.11.2230, carnitine O-acetyltransferase; 12 = Tb927.7.7500, thymine-7-hydroxylase; 13 = Tb927.1.3030, kinetoplast RNA editing ligase 2; 14 = Tb927.4.4470, GRESAG. For complete, detailed annotation and changes after 12 h RNAi induction, see Supplementary Table S6.

To interrogate the status of organelle division in TbCul-E silenced cells we used immunofluorescence against relevant organelle markers. A defect in timing of Golgi apparatus division was noted, as 1K2N cells contained a single Golgi apparatus; division of the Golgi complex precedes nuclear division, and hence suggests a general defect in coordination of division events (Fig. 6)^57^. Analysis of the whole cell proteome following 12 and 24 h of TbCul-E silencing (Fig. 7, Supplementary Tables 6, 8 and 9) revealed multiple changes. Proteins increased in silenced cells, and hence likely TbCul-E clients are hypothetical proteins Tb927.5.2120, Tb927.10.4550, Tb927.4.5160 and kinetoplastid kinetochore protein 9 (KKT9); all specific to kinetoplastids^58^, and consistent with the presence of novel kelch-domain client adaptors. There is some evidence for associations of the basal body for Tb927.5.2120, Tb927.10.4550 and Tb927.4.5160, while KKT9 silencing is also linked to a 1K2N phenotype^58^. Significantly, the 1K2N karyotype also results from silencing of ER-localised ERAP proteins^59^. Further, Tb927.10.8810, a prominent down-regulated protein in TbCul-E silenced cells, is basal body localised and kinetoplastid-specific. Finally, we note that Tb927.10.4550 has a significant role in fitness^30^, likely explaining the severe impact on TbCul-E knockdown cell viability. Hence TbCul-E appears to mediate stability and functions of a cohort of basal body/kinetochore-associated proteins involved in coordination of kinetoplastid-specific organelle replication events. These findings are consistent with roles of TbPLK, a conserved regulator of organelle replication and where knockdown of WDR1, a client adaptor, leads to defects in basal body and bilobe replication^19^.

## Discussion

Ubiquitylation is central to cellular viability and it is to be expected that the machinery encompasses both conserved and lineage-specific features, reflecting the requirement to integrate conserved functions with processes specific to a given lineage. To address divergence of ubiquitylation systems, *albeit* in a limited manner, we combined in silico and experimental analysis, using the African trypanosome as a taxonomically divergent organism and selecting cullin ubiquitin ligases for detailed study. We sought to understand how cullins have evolved across eukaryotes, if there are novel client adaptor subunits within a divergent lineage such as trypanosomes, and if function can be addressed through whole cell proteomics.

We reconstructed the evolutionary history of multiple components of cullin complexes, including cullin scaffolds, Rbx E3 ligases and client adaptors, revealing unexpected complexity. Firstly, cullin sequences that cluster within the three previously recognised ancestral cullin clades (Culα, Culβ and Culγ) include the SAR supergroup, suggesting a more ancient origin than previously noted and possibly extending to the LECA. We also identified an additional Amorphea-restricted (animals, fungi and amoeba) clade, Culδ. Further, this new topology allows us to propose that Culα initially split from the remaining cullins, followed by Culβ and Culγ, while Culδ is clearly a later innovation. The Culα clade possesses representatives from across eukaryotes, but most Excavata sequences form a unique clade Culκ. Despite challenging this topology through removal of sequences and recalculating, the Culκ clade was retained. We cannot exclude long branch attraction artefacts, suggested by similar architectures of client adaptors identified for *T. brucei* TbCul-D and *H. sapiens* CUL4a. However, we consider this evidence for extreme divergence regardless of descent and that Culκ suggests independent cullin expansion within kinetoplastids, supported by the identification of novel client adaptor protein families. This makes placement of the origin of Culβ and Culγ unresolved at this time, and is either before or after speciation of the Excavata.

**Fig. 8:**
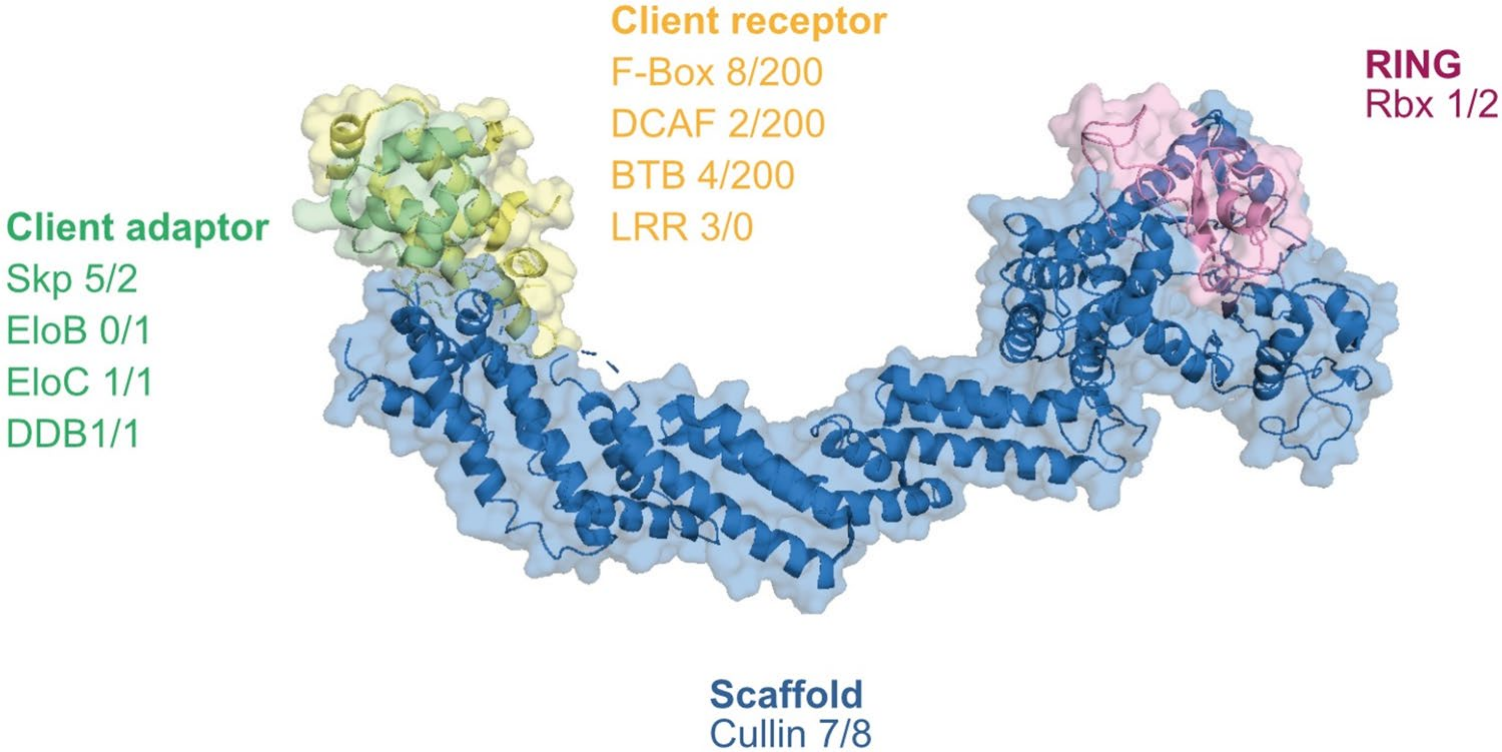
The Cullin-RING complex. The cullin protein (blue) acts as a scaffold protein to bridge the E3 ligase RING (pink) to the client adaptor (green) and receptors (yellow), which recruit and provide selectivity for the ubiquitylation substrates (scheme based on the structural model of the human Cullin 1-Rbx1-Skp1 complex; PDB: 1ldk). Beneath each subunit class are given the names and number of paralog genes in *H. sapiens* and *T. brucei*.

Building on earlier analysis we detected additional Skp-1 paralogs associated with cullin complexes (Fig. 8)^60^. These Skp-1 paralogs are mainly kinetoplastid specific, but importantly are physically associated with cullin complexes, indicating conserved roles within the ubiquitylation machinery and hence likely are adaptor proteins for recruitment of client recognition proteins. Finally, we present evidence for two additional clades of Rbx-1 related proteins, with evidence for both metazoan and kinetoplastida-specific Rbx-1 clades. The mechanistic implications of multiple Skp-1 and Rbx-1 paralogs is unclear, but does suggest the potential for independent modulation of cullin activity, and has led to the evolution of a Skp-1-containing debubiquitinase complex, TUSK, which is specific to kinetoplastids^60^.

The phylogeny of F-box, DCAF and kelch domain-containing client adaptors in kinetoplastids suggests that our affinity isolations sampled all F-box subfamilies but only a subset of DCAF and kelch family proteins. Further work is required to ascertain if these additional DCAF or kelch proteins associate with a cullin, but increases the potential size and diversity of the trypanosomatid cullin ligase repertoire. Trypanosome cullin complexes contained either Nedd8 itself or components of the neddylation machinery, indicating that trypanosomes regulate cullin ligases by a conserved mechanism and consistent with an earlier report that six trypanosome cullins are neddylated^38^. The presence of candidate client adaptor proteins possessing either kelch or LLR-domains alone, which are incorporated into F-box and BTB multi-domain adaptors in animals and fungi, suggests an evolutionary pathway where adaptor proteins grew in architectural complexity from single to multi-domain proteins.

Silencing of the cullin subunits from TbCul-A and TbCul-E complexes provides insights into the specific functions for these ligase complexes. We find ODC turnover is mediated by TbCul-A from five lines of evidence: Physical association with TbCul-A from immunoisolation, demonstration of stabilisation in TbCul-A silenced cells by both whole cell proteomics and Western blotting of genomically tagged ODC, stabilisation of ODC by MG-132 (indicating proteosome-mediated degradation) and finally, recognition of ODC with ubiquitin affinity beads. ODC is the target of eflornithine, an important trypanocide, and specificity against trypanosomes is suggested to be due to differential turnover of the human and trypanosome enzymes. Specifically, the human ODC turns over rapidly, refreshing the host ODC protein pool, while the parasite enzyme has a prolonged half-life and hence inhibition is more persistent^61^. The half-life of ODC in blood stage parasites has only been directly measured using whole cell proteomics and established as ~ 2.4 h compared with an average turnover of ~ 5.0 h in this life stage^62^; other determinations used ODC activity assays or were performed in the insect stage respectively^63,64^. Our data are consistent with the rate of turnover suggested by Tinti and coworkers^62^ and establish trypanosome ODC as an ubiquitylation client. This distinguishes the protozoal enzyme from the metazoan ortholog which is degraded without ubiquitylation, and via a mechanism involving antizyme, a protein absent from trypanosomes^65,66^. Mammalian ODC has a half-life of under 30 min *albeit* this varies greatly between cell lines and polyamine status^67,68^. We offer that our data indicate a distinct mechanism for ODC degradation in trypanosomes by a more conventional ubiquitylation route, bypassing the antizyme mechanism. Interestingly the stabilisation of TbODC by knockdown did not result in an altered EC_50_ for eflornithine (data not shown); we suggest that this may be a result of eflornithine being a suicide inhibitor and where increased resistance to eflornithine under drug selection does not result in an increase in TbODC levels^69,70^. We do not consider there to be any specific evidence for altered mRNA stability contributing to altered TbODC expression, as there is no increase to mRNA levels following TbCul-A knockdown.

TbCul-E, with lineage-specific kelch-domain client adaptors, has clear roles in organelle replication, most obviously evidenced by accumulation of aberrant 1K2N cells following silencing. Significantly, this extends to additional organelles and specifically the Golgi complex, implying a general defect in coordination of replication including basal body and kinetochore components. We suggest that coordinated turnover of components of cytoskeletal organisers provides a mechanism underpinning mitosis and which confirms and extends evidence that TbPLK is a TbCul-E substrate^19,71,72^ by identification of a larger cohort of factors likely important in control of flagellum and kinetochore replication^73^. We suggest that this final example represents a lineage-specific cullin function, incorporating novel client adaptors which control trypanosome-specific cell cycle events. This, together with our recent description of TUSK^60^, a deubiquitinase complex containing a divergent Skp-1 paralog controlling turnover of ubiquitylated surface proteins, is evidence for considerable diversification of ubiquitylation mechanisms in trypanosomes, and by extension, other eukaryotic lineages.

## Methods and materials

### Cell culture

Procyclic cells (PCF) derived from *Trypanosoma brucei* subspecies *brucei* strain Lister 427 were grown in SDM-79 JRH 57453 media (Life Technologies UK) supplemented with 7.5 mg/l of hemin (Sigma-Aldrich) and 10U/10ug/ml of penicillin/streptomycin (Thermo). Cells were maintained at densities between 5 × 10^5^ to 3 × 10^7^ cells/ml in non-vented flasks (Starlab). Cell lines transfected with the pMOT-4H plasmid^74^ were grown with 25 ug/ml of hygromycin B Gold (Invivogen).

### Genetic modifications

Cullin coding sequences were endogenously tagged using the PCR-based pMOT system^74^. OneTaq Hot Start polymerase (NEB) was used to amplify the pMOT-4H plasmid template using long oligonucleotide primers (Table S1, ThermoFisher). The PCR products were purified using PCR purification columns (Qiagen) and used for transfection. Primer sequences are given in Table S1. PCF cells at log phase were resuspended in cytomix buffer^25^ alongside 10 ug of DNA and electroporated at 1.7 kV with three pulses of 100 μs with 200 ms intervals in a BTX Gemini electroporator (ThermoFisher) using a 0.4 cm cuvette (Bio-Rad) and immediately transferred to SDM-79 media. 25 ug/mL of hygromycin B Gold (Invivogen) was added 24 h after transfection.

### Western blotting

Cells were harvested and resuspended in NuPAGE LDS sample buffer containing NuPAGE sample reducing agent (ThermoFisher). The lysate was homogenised using a sonicator (10 pulses, five seconds each) and run on NuPAGE 4–12% Bis–Tris protein gels (ThermoFisher). The iBlot 2 Dry Blotting System (ThermoFisher) was used to transfer proteins to a PVDF membrane. Membranes were blocked with 5% freeze-dried skim milk in Tris-buffered saline (TBS) buffer supplemented with 0.1% tween 20 for 30 min before overnight incubation at 4˚C with the primary antibody. After three washes with TBS of five minutes each, membranes were incubated with the secondary, HRP-conjugated antibody for one hour at room temperature prior to developing using ECL Western Blot Substrate (Pierce). Luminesence was visualised using a ChemiDoc MP Imaging System (Bio-Rad). Antibodies were used at the following dilutions: Rat anti-HA IgG1 (clone 3F10; Sigma) at 1:2000, mouse anti-Ty1 (SAB4800032, Sigma) at 1:10,000, mouse β-tubulin (clone KMX-1; Millipore) at 1:10,000. Secondary antibodies coupled to horseradish peroxidase (HRP) were anti-rat-HRP at 1:10,000 and anti-mouse-HRP at 1:10,000 (Sigma).

### Cryomilling and immunoprecipitation

Four litres of PCF *T. brucei* were grown as detailed above in 2 L roller bottles and harvested when density reached 2 × 10^7^ cells/ml. The cell pellet was washed with PBS supplemented with protease inhibitors (mini cOmplete cocktail, Roche) and snap frozen in liquid nitrogen. The frozen pellet was ground into a fine powder using a P100 mill (Retsch) as described^44^.

50 mg of cell powder were resuspended in buffer (20 mM HEPES pH 7.4, 250 mM NaCl, 1 mM Mg_2_Cl and 0.01 mM CaCl_2_ 0.1% (w/v) Brij58). After sonication, the homogenised cell suspension was centrifuged at 4 °C and 11,000 rpm for 10 min. The supernatant was incubated with Pierce anti-HA Magnetic Beads (ThermoScientific) for 2 h at 4 °C. Supernatant was discarded and the magnetic beads washed three times using resuspension buffer with reduced detergent concentration (0.01% (w/v) Brij58). The proteins bound to the beads were eluted at 70 °C for 10 min in NuPAGE loading buffer and supplemented with sample reducing agent (ThermoFisher).

### Silver staining

Visualisation of proteins in NuPAGE gels was performed using the SilverQuest Silver Staining kit (ThermoFisher) following the manufacturer’s protocol. Gels were submerged in fixative (40% ethanol, 10% acetic acid (v/v)) for 20 min before incubation for 10 min in sensitizing solution. Gels were washed in 30% ethanol (v/v), followed by incubation in the staining solution for 15 min. Before incubation in developer solution, gels were briefly washed with MilliQ Ultrapure water. Gels were imaged using a ChemiDoc MP Imaging System (Bio-Rad).

### ***RNAi with pRPa***^***iSL***^

To generate tetracycline inducible RNAi cell lines the pRPa^iSL^ was used^54^ system. Briefly, the optimal region of the cullin genes to be targeted by stem-loop RNAi was identified using RNAit2^75^, which also provided appropriate oligo primers (Table S1). PCR products were purified and digested using the appropriate enzymes (NEB) as described^54^, before using T4 DNA ligase (NEB) to insert it into the linearized pRPa^iSL^ MCS1/2 plasmid. The complete pRPa^iSL^ with both PCR inserts was digested with AscI and the fragment of interest was cleaned-up using a gel extraction kit (Qiagen) to be used for transfection.

### RNA extraction and quantitative 

*RT-PCR:* RNA extraction was performed using the RNeasy Kit (Qiagen). Briefly, 5 × 10^7^ cells were harvested (800 g, 24 °C, 10 min) and washed twice with PBS before lysis using the RLT buffer from the kit. The lysate was mixed with 70% (v/v) ethanol in DEPC-treated water (Invitrogen) and processed essentially following the manufacturer instructions. Reverse transcription and quantitative PCR was performed using the Luna Universal One-step RT-qPCR Kit (NEB) using 1 μg of RNA per reaction. Reactions were carried out in a QuantStudio 3 real time PCR system (ThermoFisher) and included initial denaturation at 95 °C for 1 min followed by 40 cycles of 95 °C for 10 s and 60 °C for 30 s with a signal read at the end of each cycle plus a final melting curve to check fidelity from 60 to 95 °C, with a signal read every 1 °C. The data were normalised against the *T. brucei β*-tubulin transcript. Fold changes in gene transcription were calculated using the Ct method, normalised to the parental strain *T. brucei* 2T1 and displayed as relative quantity.

### Proteomics

Affinity capture eluates from the magnetic beads were loaded onto NuPAGE Bis–Tris 4–12% gradient polyacrylamide gels as described previously^76^. Briefly, samples were run ~ 1 cm into the gel and cut using a virgin scalpel. Slices were subjected to tryptic digest and reductive alkylation using routine procedures and eluted peptides analysed by LC–MS/MS on an UltiMate 3000 RSLCnano System (Thermo Scientific) coupled to a LTQ OrbiTrap Velos Pro mass spectrometer (Thermo Scientific). TbCul-E RNAi samples were prepared label-free at two timepoints (12 and 24 h after induction with 1ug/ml tetracyclin). Uninduced cells were used as controls. Cells were washed with PBS containing complete protease inhibitors (Roche), extracted with 1 × NuPAGE sample buffer and sonicated. Lysates containing 1 × 10^7^ cells were fractionated on a NuPAGE Bis–Tris 4–12% gradient polyacrylamide gel (Thermo) under reducing conditions. The approximately 30 mm migration portion was cut into three slices that were subjected to tryptic digest and reductive alkylation using routine procedures. The eluted peptides were then analyzed by liquid chromatography-tandem mass spectrometry (LC-MS2) on a ultimate3000 nano rapid separation LC system (Dionex) coupled to a Q Exactive HF (Thermo-Fisher Scientific).

TbCul-A RNAi samples were prepared using SILAC. HMI-9 for SILAC was prepared as described previously^77^. Either normal L-Arginine and L-Lysine (HMI11-R0K0), or L-Arginine − ^13^C_6_ and L-Lysine ^4,4,5,5-2^H_4_ (HMI11-R_6_K_4_) (Cambridge Isotope Laboratories) were added at 120 μM and 240 μM respectively. Cells with induced TbCul-A RNAi were grown in parallel with uninduced cells, in the presence of HMI11-R_0_K_0_ or HMI11-R_6_K_4_, respectively. Cultures in logarithmic growth were mixed after 24 and 48 h, immediately harvested by centrifugation, washed twice with PBS containing protease inhibitors (Roche) and resuspended in Laemmli buffer containing 1 mM dithiothreitol. Samples were generated in triplicate and one label swap was included. Samples were sonicated and aliquots containing 5 × 10^6^ cells separated on a NuPAGE bis–tris 4–12% gradient polyacrylamide gel (Thermo). The sample lane was divided into eight slices that were excised from a Coomassie stained gel, destained, then subjected to tryptic digest and reductive alkylation. The eight fractions obtained from SDS-PAGE were subjected to LC–MS/MS on a UltiMate 3000 RSLCnano System (Thermo Fisher Scientific) coupled to an Q Exactive HF (Thermo Fisher Scientific) mass spectrometer.

Mass spectra were processed in MaxQuant^78^ v1.5. or 1.6.1.0 (Cul-E RNAi) searching the *T. brucei brucei* 927 annotated protein database (release 42) from TriTrypDB^52^. Affinity capture and Cul-E RNAi spectra were processed using the intensity-based label-free quantification (LFQ). For TbCul-A RNAi, SILAC ratios were calculated using only peptides that could be uniquely mapped to a given protein. False discovery rates (FDR) of 0.01 were calculated at the levels of peptides, proteins and modification sites based on the number of hits against the reversed sequence database. Perseus^79^ was used for statistical analysis. For TbCul-E RNAi and affinity capture, LFQ values were log_2_ transformed, and missing values imputed from a normal distribution of intensities around the detection limit of the mass spectrometer. A Student’s *t*-test (using the ratio of the difference in sample group means over the pooled standard error of both sample groups) was used to compare the LFQ intensity values between replicate sample groups. These were triplicate sample groups for TbCul-E RNAi and, for the affinity capture, triplicate bait samples (3xHA tagged Cullin) and seven replicates of an untagged control (WT parental cells). − log_10_ p-values were plotted versus *t*-test difference to generate volcano plots. Potential interactors were classified according to their position in the volcano plot, applying cutoff curves for “significant class A” (SigA; FDR = 0.01, s0 = 0.1), “significant class B” (SigB; FDR = 0.05, s0 = 1). The cutoff is based on the false discovery rate (FDR) and the artificial factor s0, controlling the relative importance of the *t*-test *p*-value and difference between means (at s0 = 0 only the p-value matters, whereas at nonzero s0, the difference of means contributes).

### Bioinformatics

*H. sapiens* cullin proteins (Uniprot ID: Q13616, Q13617, Q13618, Q13619, Q13620, Q93034, Q14999 and Q8IWT3) were used as queries against the genome of *Trypanosoma brucei brucei* TREU927 in a BLASTp^80^ search (BLOSUM62, Gap existence: 11, Gap extension: 1, Conditional compositional score matrix adjustment) to retrieve cullin genes from *T*. *brucei*. The *Trypanosoma spp.* hits were then used as queries in a BLASTp search against their own genome and the top 10 hits considered for further analyses. The selected hits were used as queries in reverse BLASTp searches against curated lists of organisms of the Archaeplastida, SAR, Excavata, Amoebozoa and Ophistokonta. As cut-off, a maximum of five hits per organisms per query with an e-value < 0.001 and coverage > 30% were recorded. Coverage was defined by either a calculated coverage (addition of non-overlapping local alignments with gaps removed) or the BLAST qcovs parameter (query coverage per subject). These hits, as well as those identified using subunit 2 of the anaphase promoting complex (Uniprot ID: Q9UJX6) as query, were aligned using MUSCLE (version 3.8.1551)^81^ and edited with alncut (version 1.06)^82^ only allowing 25% or fewer of the sequences to have a gap in every given amino acid position. FastTree (version 2.1.10)^83^ was used to generate initial phylogenetic reconstructions of the cullin family for each of the Eukaryota supergroups. Using ScrollSaw^41^ two protein entries per clade per tree were selected, pooled and aligned as described above to create a pan-eukaryotic cullin tree using FastTree, PhyML 3.0^84^ and MrBayes^85^. For PhyML, 1000 bootstrap and the LG model were used while FastTree was used with default settings. MrBayes was perfomed using the CIPRES Science Gateway with BEAGLE and the following block was added to the input files: nrun = 2 nchains = 8 lset rates = gamma Ngammacat = 4 nucmodel = protein code = universal; prset aamodelpr = mixed; mcmcp ngen = 8,000,000 relburnin = yes burninfrac = 0.25 printfreq = 100,000 samplefreq = 1000 diagnfreq = 1000 nchains = 8 savebrlens = yes;. MrBayes analysis was run to convergence.

Phylogenetic analysis of Skp and Rbx did not require ScrollSaw, and reverse BLASTp searches against a curated selection of eukaryotes from Archaeplastida, SAR, Excavata, Amoebozoa and Ophistokonta were used to identify orthologs and paralogs in each supergroup. The queries used were *H. sapiens* SKP1, RBX1 and RBX2 as well as ELOB and APC11 as outgroups (Uniprot ID: P63208, P62877, Q9UBF6, Q15370, Q9NYG5). Other Skp and Rbx candidates identified from the immunoprecipitation of cullin complexes in *T. brucei* were also used as query (Tb927.10.11610, Tb927.9.8570, Tb927.11.13330, Tb927.10.14310, Tb927.11.6130). Again, the cut-off was a maximum of five hits per organisms per query with an e-value < 0.001 and coverage > 30% (as either calculated coverage or qcovs) were recorded. For each of the trees, the hits were aligned and edited as described above and the phylogenetic trees were reconstructed using FastTree, PhyML and MrBayes using the same parameters as before. The reverse BLASTp searches across eukaryotes, the alignment of sequences and generation of trees with FastTree were performed using in house automated Python and Bash scripts^86^. Trees were visualised, annotated and coloured using FigTree^87^ Fasta files for the sequences used to generate each phylogenetic tree and a list of species for each are provided as a supplementary data archive.

Reconstruction of substrate receptor families was generated using orthology BLASTp searches against a curated list of taxa with *T. brucei* proteins as query. Default settings were applied as above and the top five hits with an e-value < 0.0001 and a coverage (qcovs) > 70% were recorded. Hits were aligned using MUSCLE and edited with alncut as above. FastTree, PhyML and MrBayes were used as above to perform phylogenetic reconstructions. Reverse BLASTp searches using either *H. sapiens* or *A. thaliana* proteins as query were performed in the same manner.

### Immunofluorescence microscopy

*T. brucei* cells were harvested and washed twice using Dulbecco’s PBS (Thermo), then fixed using 3% paraformaldehyde (Thermo) at 37 °C for 10 min and resuspended in Dulbecco’s PBS. Cells were allowed to settle onto a poly-lysine slide (VWR) for approximately 45 min at room temperature. The cells were then permeabilized using 0.2% Triton X-100 for 10 min and incubated for 1 h with 20% FBS. Primary antibodies were incubated at 4 °C overnight, followed by incubation with secondary antibodies for 1 h at room temperature. Primary antibody concentrations were rabbit anti-GRASP (from M. Ferguson, University of Dundee) at 1:1000 and secondary antibody (Thermo) anti-rabbit Alexa-568 at 1:1000. MitoTracker (Thermo) was added to cultures to a final concentration of 1 nM 5 min before harvesting. Vectashield with 4,6-diamidino-2-phenylindole (DAPI; Vector Laboratories, Inc.) was applied to slides before covering them with a coverslip (WVR). Cells were visualised using a Zeiss Axiovert 200 microscope with an AxioCam camera and ZEN Pro software (Carl Zeiss, Germany) and images acquired as z-stack of 0.26 μm. Images were generated using OMERO (https://www.openmicroscopy.org/omero/)^88^.

## Supplementary Information

Supplementary Information 1.

Supplementary Information 2.

Supplementary Information 3.

Supplementary Information 4.

Supplementary Information 5.

Supplementary Information 6.

Supplementary Information 7.

## Data Availability

Proteomics data associated with these analyses have been deposited at the Pride Proteomics database at https://www.ebi.ac.uk/pride with accession number: PXD042022.
