# Supplementary material for: Evolution, composition and functions of cullin E3 ubiquitin ligases in trypanosomes
Source: Sci Rep. 2025 Dec 18;16:2285. doi: 10.1038/s41598-025-32077-9 (PMC12816148; doi:10.1038/s41598-025-32077-9)
Supplement: Supplementary file 1 — Supplementary Information 1. [file 41598_2025_32077_MOESM1_ESM.docx]

**Supplementary material for:**

**Evolution, composition and functions of cullin E3 ubiquitin ligases in trypanosomes**

Ricardo Canavate del Pino^1^, Martin Zoltner^1,2^, Erin R. Butterfield^1^ and Mark C. Field^1,3*1^

^1^School of Life Sciences, University of Dundee, Dundee, DD1 5EH, UK, ^2^Charles University in Prague, Faculty of Science, Department of Parasitology, Průmyslová 595, Vestec 252 42, Czechia, and ^3^Institute of Parasitology, Biology Centre, Czech Academy of Sciences, České Budějovice, Czechia.

**Supplementary figure legends**

**Figure S1: Validation and interactome of tagged *T. brucei* cullins.** Panel A: Western blotting against the human influenza hemagglutinin (HA) epitope endogenously fused to the cullin genes of *T. brucei*. Note the low abundance of TbCul-C and TbCul-F. No clones successfully expressing TbCul-B were obtained. Molecular weight markers are shown at left in kDa. All samples were separated in the same polyacrylamide gel but the chemiluminescence in the TbCul-C and TbCul-F lanes have been contrast-enhanced over the regions indicated with dotted lines. P is parental line. All lanes contained ~1 x 10^7^ cell equivalents. Panel B: Silver stained SDS-PAGE of eluates from affinity purified 3xHA::cullin complexes from *T. brucei* alongside their interactors. Each band specific to the tagged cell line (i.e. not found in the control) has been labelled based on the predicted molecular mass of interactors identified through mass spectrometry, with the migration position of the tagged cullin based on panel A ndicated with a white circle (Figure 2). Lanes are labelled for the cullin isolated (see text), with “P” corresponding to untagged procyclic cells (negative control). Migration positions of molecular weight markers are indicated at left in kDa and for immunoglobulin heavy and light chains at right as IgH and IgL respectively.

**Figure S2: The Skp-1 family is expanded in Excavata.** Phylogenetic reconstruction of the SKP1 family of protein adaptors in Eukaryota. Skp1.1 is present in all eukaryotic supergroups while Excavata has four additional members: Skp1.2, Skp1.3, Skp1.4 and Skp-Z. The five clades are indicated with black bars and each taxon is coloured based on the supergroup to which it belongs. Elongin factor B (EloB), that acts as protein adaptor in some *H. sapiens* cullin-RING complexes, was used as an outgroup to root the tree. Support of nodes is indicated with dots, closed or open depending on the level of confidence as shown on the bottom left. *T. brucei* sequences are annotated with their TriTrypDB accession numbers while all others by species.

**Figure S3: The F-box family in Kinetoplastida.** Black bars mark each of the clades and stars indicate proteins co-precipitated with TbCul-A. The tree is rooted at midpoint and taxa coloured coded as indicated. The Bayesian posterior probability is given for each node. *Trypanosoma grayi* is coloured as an American trypanosome due to lacking the variant surface glycoprotein antigenic variation system of African trypanosomes.

**Figure S4: DCAF substrate adaptors in Kinetoplastida.** Stars mark the two proteins identified in the immunoprecipitation of TbCul-D. Clades are indicated by black bars and the Bayesian posterior probability is indicated for each node. All sequences are annotated as TriTrypDB accessions. The tree is pseudo-rooted at midpoint and taxa colour coded as indicated.

**Figure S5: Kelch protein interactors of TbCul-E in Kinetoplastida.** The stars mark the four proteins identified in the immunoprecipitation of TbCul-E. Clades are indicated by black bars and the Bayesian posterior is indicated for each node. All sequences are annotated as TriTrypdb accessions. The tree is pseudo-rooted at midpoint and taxa coloured coded as indicated.

**Figure S6: Sequence similarity of novel Rbx proteins.** A cluster pairwise alignment is shown as an output from Jalview. Note that despite large indels the remaining sequences are of clear close similarity.

**Figure S7: RT-qPCR validation of RNAi.** RNA was extracted at 24 hours from cells induced or non induced for TbCul-A and TbCul-E knockdowns. RT-qPCR was conducted as indicated in methods. Data are from three replicate extractions for each analysis and error bars indicate the standard deviation.

**Figure S8: Growth curve for cells induced for TbCul-E RNAi.** Cells were monitored and counted at the indicated times for three separate cultures for each condition. Error bars indicate the standard deviation.

**Supplementary figure 9: Larger scans of Western blots in figure 5.** Panel A: Elution of TbODC from ubiquitin-recognising beads. TbODC with a Ty1-tag endogenously fused to its C-terminus was captured by UbiQapture-Q beads after incubation with cell lysate. WT; parental line, antibody against tubulin was used as a loading control. The intensity of TbODC-10xTy1 detected from immunoprecipitation and from the total fraction increased following induction of TbCul-A RNAi for 24 hours with tetracycline. Note positions of coelectrophoresed molecular eight markers (lane L). Panel B: Degradation of TbODC is mediated by the proteasome. Left; turnover of TbODC as measured following inhibition of protein synthesis with cyclohexamide (CHX) and right; turnover of TbODC as measured following inhibition of protein synthesis and proteasome activity with MG132. Blot is representative of four replicates. Numbers above lanes indicate time post RNAi induction in hours. Antibody against tubulin was used as a loading control.

**Supplementary tables**

**Table S1: Sequences of primers used in this work.** Sequences are written 5’ to 3’ and the plasmid system for which each primer was used indicated. Bold sequences are shared with pMOT and upper/lower to differentiate base triplets.

**Table S2: Identification of cullin complex components.** Proteins significantly enriched in the immunoprecipitation of the cullin complexes. In the columns, the gene IDs for each protein identified, their names and the values from a statistical t-test used in the volcano plot. For TbCul-A, the shadowed column shows an F-box protein identified in the IP that did not pass the cut-off set with a false discovery rate of 5% and a minimum fold change S0 at 1.0.

**Table S3: Proteins identified and shown in volcano plots.** Data for proteins called out in Figure 4.

**Table S4: Species used in phylogenetic analysis.**

**Table S5: Proteins affected by the knockdown of TbCul-A.** SILAC ratios and transformed p-values used to visualize in a volcano plot the effect of TbCul-A RNAi on the proteome of bloodstream *T. brucei* cells. ERO1, endoplasmic reticulum oxidoreductin 1; GRESAG, gene related to expression site associated genes, KIN2B, Kinase II B; ODC, ornithine decarboxylase; SAMdc, S-adenosyl decarboxylase.

**Table S6: Proteins affected by the knockdown of TbCul-E.** SILAC ratios and transformed p-values used to visualize in a volcano plot the effect of TbCul-E RNAi on the proteome of bloodstream *T. brucei* cells.

**Table S7: Proteins affected by the knockdown of TbCul-A.** Label-free quantification (LFQ) ratios and transformed p-values used to visualize in a volcano plot the effect of TbCul-A on the proteome of bloodstream *T. brucei* cells.

**Table S8: Proteins affected by the knockdown of TbCul-E after 12 hours.** Label-free quantification (LFQ) ratios and transformed p-values of the effect of TbCul-E knockdown on the proteome of bloodstream *T. brucei* cells. ESAG3, expression site-associated gene 3; VSG, variant surface glycoprotein; ISG65, invariant surface glycoprotein 65 kDa; ZC3H47, Zinc finger CCCH domain-containing protein 47.

**Table S9: Proteins affected by the knockdown of TbCul-E after 24 hours.** Label-Free quantification (LFQ) ratios and transformed p-values used to visualize in a volcano plot the effect of TbCul-E RNAi on the proteome of bloodstream *T. brucei* cells. KREL2, kinetoplast RNA-editing ligase 2; ESAG11, expression site-associated gene 11; LEM3, ligand effector modulator 3; VSG, variant surface glycoprotein; KKT9, kinetoplastid kinetochore protein 9; GRESAG, gene related to ESAG.

**Supplementary data archive:** Fasta sequences for predicted proteins included in the phylogenetic analysis of cullins, Skp orthologs and Rbx orthologs.
