# Supplementary figures and images for: Evolution, composition and functions of cullin E3 ubiquitin ligases in trypanosomes
Source: Sci Rep. 2025 Dec 18;16:2285. doi: 10.1038/s41598-025-32077-9 (PMC12816148; doi:10.1038/s41598-025-32077-9)

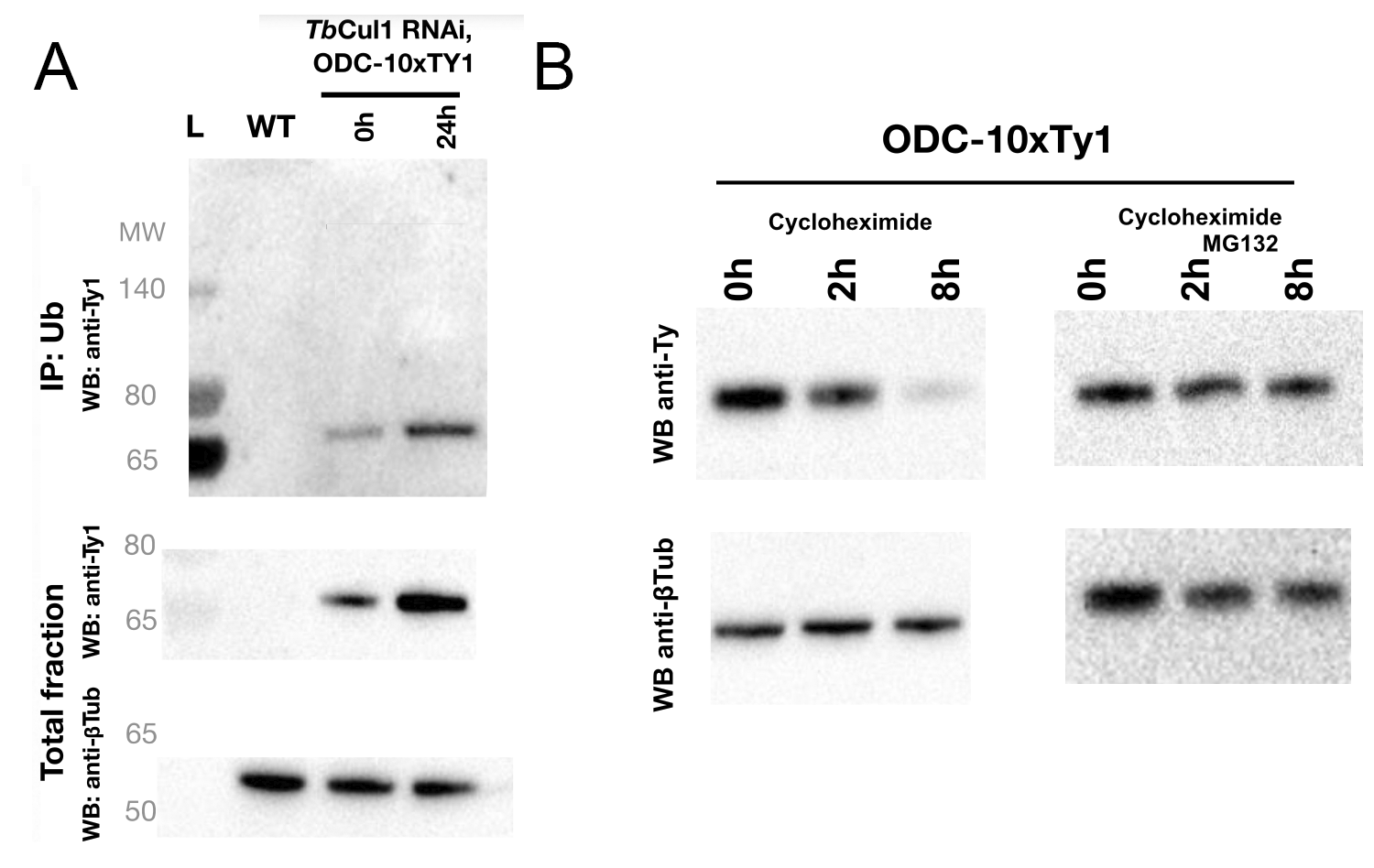

Supplement: Supplementary file 2 — Supplementary Information 2. [file 41598_2025_32077_MOESM2_ESM.tif]
